# Toxic metabolite profiling of *Inocybe virosa*

**DOI:** 10.1038/s41598-020-70196-7

**Published:** 2020-08-13

**Authors:** S. Sai Latha, Naveen Shivanna, Mahadeva Naika, K. R. Anilakumar, Ankur Kaul, Gaurav Mittal

**Affiliations:** 1 Department of Food Quality and Assurance, Defence Food Research Laboratory, Defence Research and Development Organisation, Ministry of Defence, Govertment of India, Siddarthanagar, Mysore, 570011 India; 2 Institute of Nuclear Medicine and Allied Sciences, Defence Research and Development Organisation, New Delhi, 110054 India

**Keywords:** Biochemistry, Biophysical methods

## Abstract

Wild mushroom foraging involves a high risk of unintentional consumption of poisonous mushrooms which is a serious health concern. This problem arises due to the close morphological resemblances of toxic mushrooms with edible ones. The genus *Inocybe* comprises both edible and poisonous species and it is therefore important to differentiate them. Knowledge about their chemical nature will unambiguously determine their edibility and aid in an effective treatment in case of poisonings. In the present study, the presence of volatile toxic metabolites was verified in *Inocybe virosa* by gas chromatography. Methyl palmitate, phenol, 3,5-bis (1,1-dimethyl ethyl) and phytol were the identified compounds with suspected toxicity. The presence of the toxin muscarine was confirmed by liquid chromatography. The in vitro study showed that there was negligible effect of the digestion process on muscarine content or its toxicity. Therefore, the role of muscarine in the toxicity of *Inocybe virosa* was studied using a bioassay wherein metameters such as hypersalivation, immobility, excessive defecation, heart rate and micturition were measured. Administration of muscarine resulted in an earlier onset of symptoms and the extract showed a slightly stronger muscarinic effect in comparison to an equivalent dose of muscarine estimated in it. Further, the biological fate of muscarine was studied by pharmacokinetics and gamma scintigraphy in New Zealand white rabbits. Significant amount of the toxin was rapidly and effectively concentrated in the thorax and head region. This study closely explains the early muscarinic response such as miosis and salivation in mice. By the end of 24 h, a relatively major proportion of muscarine administered was accumulated in the liver which stands as an explanation to the hepatotoxicity of *Inocybe virosa.* This is one of the rare studies that has attempted to understand the toxic potential of muscarine which has previously been explored extensively for its pharmaceutical applications.

## Introduction

Wild mushroom foraging involves a high risk of unintentional consumption of poisonous mushrooms which is a serious health concern. This problem arises due to the close morphological resemblance of toxic mushrooms with edible ones. The genus *Inocybe* also comprises both edible and poisonous species and it is therefore important to differentiate them. The species *Inocybe virosa* which is the focus of the present study is closely allied to *Inocybe cutifracta* which is an edible mushroom. The taxonomic classification of a mushroom species under the genus *Inocybe*, generally raises a suspicion about its edibility. This inevitable caution stems from the literature which abounds in cases of poisonings due to *Inocybe* mushrooms. Some of the poisonings reported have been associated with *Inocybe fastigiata*^1,2^, *I. tristis*^3^, *I. anterospora*^4^, *I. aeruginascens*^5^ and *I. patouillardii*^6^. Species such as *I. asterospora, I. gobeyi, I. napipes, I. repanda, I. radiata, I. rimosa * show psychotropic effects and classified as neurotoxic. Most of the *Inocybe* species are toxic or apparently toxic^7^. Knowledge about their chemical nature will unambiguously determine their edibility and aid in an effective treatment in case of poisonings. A few studies have been carried out on *Inocybe* species to identify their toxic principles: Jensen et al., 2006 reported aeruginascin, (a trimethyl ammonium analog of psilocybin) in *Inocybe aeruginascens*^8^; Zhao et al.^9^ associated the toxicity of *Inocybe umbrinella* to the presence of lectins and biogenic amines were identified in *I. patouillard*^10^. The toxin muscarine was determined in *I. cinnamomea, I. geophylla*, and *I. lacera*^11^ and its isomer epi-muscarine was identified in *I. geophylla*^12^. Gas Chromatography is one of the techniques for toxicological assessment of volatile toxicants while for non-volatile toxicants it is Liquid Chromatography^13^. In the present study, characterization of *Inocybe virosa* was done by both gas chromatography and liquid chromatography to identify and quantify the suspected metabolites. Also, the role of muscarine in toxicity of *Inocybe virosa* was confirmed by both in vitro and in vivo studies. Further, the pharmacokinetics of muscarine was studied to deepen the understanding of its toxicity.

## Materials and methods

### Sample collection

Mushroom samples were collected from a forest locality in Trivandrum district of Kerala state, India, during monsoon months of the year 2014. The mushrooms were scientifically studied first for their macroscopic and then for its microscopic appearances and finally identified by authenticated taxonomic solutions^14^. The specimens were deposited in Mycological Herbarium of Jawaharlal Nehru Tropical Botanic Garden and Research Institute, Trivandrum [TBGT]. A brief taxonomic description of the mushroom is given below:

***Inocybe virosa*** Pradeep CK, Vrinda KB and Matheny (voucher no.TBGT15403).

**Pileus** 3–9 cm diameter, convex expanding plane, broadly umbonate; surface brownish orange with a yellowish brown centre, radially appressed fibrillose, rimose towards the margin; Lamellae adnexed, marble white becoming brownish with age. **Stipe** 4–10 cm × 4–11 mm, central, cylindric, equal, fistulose; surface yellowish white, longitudinally fibrillose striate. Context dull white to pale buff. **Basidiospores** 6.5–7.5 × 4.5–5 µm, ellipsoid with smooth yellowish brown lightly thickened wall. Basidia 20–31 × 6–11.5 µm, clavate, 4–spored. Cheilocystidia 18–38 × 15–20 µm, clavate thin-walled. Pileipellis a repent epicutis. Clamp connections present.

### Mushroom extract preparation^15^

The collected specimens were first dried at 27 ± 3 °C until completely dehydrated and then stored at 4 °C in an air-tight container. The mushrooms were powdered and then extracted using 50% (V/V) ethyl alcohol. Approximately 100 mL of ethanol was added to 5 g mushroom and extraction was done by continuous shaking at 100 rpm for 12-15 hrs overnight. This process were repeated 4–5 times, the filtrates of each extraction were then pooled and subjected to rotary evaporation using vacuum. After removal of alcohol it was dried using lyophilizer to obtain alcohol-free powdered extract. This extract thus obtained was used for further studies.

### GC–MS analysis

GC–MS analysis of the hydro-ethanolic extract of *Inocybe virosa* was performed using the method followed by Sai Latha et al.^15^. Agilent GC 6890 N JEOL GC Mate II with Ion Trap gas-chromatography equipped with HP5 capillary column (30 m × 0.32 mm; coating thickness 0.25 μm), interfaced with Agilent 240 MS Ion Trap mass detector was used for this analysis. The analytical conditions were as follows: injector temperature—20 °C; transfer line temperature—250 °C; oven temperature programmed from 50 °C to 250 °C at 10 °C /min; helium as the carrier gas at 1 mL/min; ionization voltage—70 eV; ion source temperature—250 °C; interface temperature—250 °C; mass range—50–600 mass units. The identification of components was performed by comparison of their linear retention indices and by matching against commercial mass spectra libraries from NIST and MS literature data.

### RP-HPLC and LC–MS analysis

The *Inocybe virosa* mushroom as well as its hydro-ethanolic extract were analysed for the presence of muscarine using Reverse Phase-High Pressure Liquid Chromatography (RP-HPLC).The samples were subjected to extraction procedure followed by Yoshioka et al.^16^ with slight modifications. 0.2 g of finely powdered samples was homogenized with 2.5 mL 0.5% formic acid in methanol and sonicated for about 5 min. This mixture was centrifuged at 1,000 ×*g* for 5 min and the supernatant was collected. The final volume was adjusted to 5 mL using 50% methanol (V/V). For the clean-up, 1 mL was loaded to Oasis HLB cartridge preconditioned with methanol. The first 0.5 mL of the elute was discarded and the remaining was collected for analysis. Muscarine was determined using JASCO HPLC system (Japan) consisting of quaternary pump and PDA (Photo Diode Array) detector. Separation was carried out on a HiQSiL Reverse phase C_18_ column (250 mm × 4.6 mm, particle size 5 µm). The absorbance was monitored at 235 nm and the results were analysed using LCNET software. A gradient elution system using the mobile phase A (5 mM K_2_HPO_4_ buffer, pH-2.5 prepared in Milli-Q water) and mobile phase B (Methanol, HPLC grade) at a flow rate of 0.6 ml/min. The gradient program was as follows: (1) 0 min-100% A, (2) 3 min-40% A, (3) 9 min-40% A, (4) 12 min-100% A. The muscarine content was estimated in the mushroom extract by using standard muscarine (Sigma-Aldrich, Chemical purity ≥ 98%). The chromatogram of RP HPLC of standard is shown in Supplementary Fig. S1.

An Agilent HPLC 1260 Series connected with Shimadzu LC–MS 2020 system and an Agilent Eclipse plus column (4.6 × 250 mm, 5 µm) was used for MS analysis of the samples. The same gradient program was employed for the analysis as in HPLC with a slight modification of replacing K_2_HPO_4_ buffer with 0.1% formic acid as solvent A. The specifications of MS analysis were as follows: ESI-Positive ionization; Desolvation line temprature−250 °C; Heat block temperature−200 °C; Scan-50 m/z to 500 m/z; Scan speed—455u/s; Detector Voltage—0.8 kV; Mobilizing gas flow—1.5 L/min.

### Cytotoxicity evaluation of *Inocybe virosa*

#### Cell lines

The immortalized cell lines, NCM460 and Chang liver cell line were both obtained from National Centre for Cell Sciences (NCCS), Pune, India and grown at 37 °C in a humidified atmosphere of 5% CO_2_ using MEM (Minimum Essential Media), supplemented with 10% FBS and antibiotic solution. About 80% confluent cells were used for the assays. The analysis was carried out in triplicates.

#### Cell viability

The in vitro cytotoxicity of the mushroom extract was assessed on NCM460 and Chang liver cell lines using the dye 3-(4,5-Dimethylthiazol-2-yl)-2,5-diphenyltetrazolium bromide (MTT). The cells (100 μL; 1 × 10^5^ cells/mL) were seeded into 96 well plates and incubated for 24 h. The medium was then removed from the wells and replaced with 100 μL filter sterilized medium containing the mushroom extract. The cells were incubated with different concentrations (0–2.5 mg/mL) of the extract for the next 24 h. MTT (100 μl; 0.5 mg/mL) was added to each well and further incubated for 2 h. Subsequently, the medium was removed and DMSO (100 μL) was added. The absorbance was recorded at 570 nm^17^ and cell viability was expressed with respect to control. The toxic effects of the extract on cell morphology were also noted and photographed.

### Evaluation of in vivo oral toxicity of *Inocybe virosa*

#### Animals and housing conditions

Female Balb/c mice of body weight 25–30 g, from the Central Animal Facility of the laboratory (Defence Food Research Laboratory), were used for the study. Six animals per cage were housed in standard sized cages with corn cob bedding and maintained under controlled 12 h light/dark cycle, optimum conditions of temperature and relative humidity (23 ± 2 °C, 40–60% humidity). Potable water and pellet diet were provided ad libitum to the animals. The experimental procedures were performed in accordance with the guidelines of CPCSEA (Committee for the Purpose of Control and Supervision on Experiments on Animals)and approved by IAEC (Institutional Animal Ethics Committee), Defence Food Research Laboratory, DRDO (Ref no. DFRL/IAEC/01/2015). The study design followed OECD guidelines TG 423 (2001) for assessing acute toxicity and TG 407 (2008) for sub-acute toxicity.

#### Dose formulation and administration

The mice were randomly divided into 3 groups (n = 6). The animals were fasted for 2 h prior to the administration of mushroom extract, and control animals were administered water in place of the extracts. For acute toxicity study, a single dose of each mushroom extract was administered to two groups of animals, one at 250 mg/kg body weight (bwt) and the other at 500 mg/kg bwt. The animals were observed for any abnormal behavioural responses and body weight changes. Three animals of each group were sacrificed after 24 h and the remaining three animals were sacrificed on day 14 under anesthetic conditions. Blood was drawn through cardiac puncture and analysed for haematological parameters and biochemical parameters were analysed in the serum. The organs were collected for histopathological analysis.

#### Hematological analysis

For hematological analysis, the red blood cell (RBC) count, hemoglobin concentration (Hgb), hematocrit (Hct), platelets (PLT) and white blood cell (WBC) count were measured in the uncoagulated blood using an automated hematology analyzer, Sysmex KX-21 (Tranasia Bio-medicals Pvt.Ltd., India).

#### Blood biochemical analysis

Biochemical parameters in serum were analyzed to assess the liver and kidney functioning using the commercially available kits from Agappe. The liver function tests (LFTs) included the estimation of enzyme activities such as aspartate transaminase (AST), alanine aminotransferase (ALT), alkaline phosphatase (ALK), and the total bilirubin (TBIL) content. The kidney function tests (KFTs) included the estimation of creatinine (CRE), blood urea nitrogen (BUN), and total protein (TPR).

#### Histopathological studies

The organs viz., brain, kidney, liver, small and large intestine were excised on sacrificing the animals. The tissues were gently rinsed in phosphate buffer (pH 6.8) and were fixed with 10% neutral buffered formalin solution. The tissues were then embedded in paraffin wax, sectioned using a microtome and mounted onto glass slides. The paraffin was cleared and this was followed by staining the tissue section with hematoxylin and eosin. The cross sections of the tissues were observed under a compound light microscope at 20 × objective.

### Toxicity of muscarine

#### In vitro digestion of muscarine

In vitro digestion of *Inocybe virosa* extract was carried out using the model proposed by Soler‐Rivas et al.^18^. 10 g of the extract was mixed with 5 mL of water and heated to boiling. The sample was cooled and mixed with 1 mL of healthy volunteer’s saliva and 1 mL of phosphate buffer (0.08 M, pH 6.7). The pH was then set to 2 with 6 M HCl and pepsin was added (5 mg per gram homogenate). The mixture was then incubated for 2 h at 37 °C with slow stirring at 60 rpm in a rotary shaker. The sample was subjected to further digestion by adding 2 mL of pancreatic solution (4 mg pancreatin and 25 mg bile salts per mL of 0.1 M NaHCO_3_) and the pH was adjusted to 7.5 with NaOH. To the digested samples, 1 g of NaHCO_3_ was added for maintaining the pH. The mixture was incubated again for 2 h at 37 °C with slow stirring at 60 rpm.

On complete digestion of the sample, cytotoxicity of the digestate was assessed on a human epithelial colorectal cell line, Caco2 obtained from NCCS, Pune. The cells were grown and maintained using Minimum Essential Medium (MEM) (Sigma-Aldrich) supplemented with 10% foetal bovine serum (HiMedia), 1% penicillin–streptomycin (Sigma-Aldrich), 1.5 g/L Sodium bicarbonate and 110 mg/L Sodium pyruvate (Sigma-Aldrich) at 37 ºC in a humidified atmosphere containing 5% CO2. The cells (100 μL; 1 × 10^5^ cells/mL) were seeded into 6 well plates and incubated for 24 h. On the adherence of cells, medium was removed from the wells and the wells in triplicates were incubated overnight with 500 μL filter sterilized medium containing the mushroom extract and digestate. The cytotoxic effect was evaluated using the dye 3-(4,5-dimethylthiazol-2-yl)-2,5-diphenyltetrazolium bromide (MTT). MTT (100 μl; 0.5 mg/mL) was added to each well and further incubated for 2 h. Subsequently, the medium was removed and DMSO (100 μL) was added. The absorbance was recorded at 570 nm^17^ and cell viability was expressed with respect to control. The toxic effects of the extracts on cell morphology were also noted and photographed. A set of cells in triplicates were also exposed to the mixture of digestive enzymes used for the in vitro digestion. To verify the effect of digestion on muscarine, the digestate was analysed for the toxin concentration using RP-HPLC method detailed in “RP-HPLC and LC–MS analysis” section.

#### In vivo muscarinic response

The experimental procedures were performed in accordance with the guidelines of CPCSEA (Committee for the Purpose of Control and Supervision on Experiments on *Animals) *and approved by IAEC (Institutional Animal Ethics Committee), Defence Food Research Laboratory, DRDO (Ref no. DFRL/IAEC/01/2015). *Inocybe virosa* (IV) extract at 500 mg/kg bwt was orally administered to mice weighing about 25–30 g (n = 3). Another set of mice were administered an equivalent dose of standard muscarine (MC) (i.e., 4.5 mg/kg bwt muscarine corresponds to the amount present in 500 mg/kg bwt of IV (*Inocybe virosa*) extract based on the HPLC analysis) and water was administered to the control group. The behavioural pattern of the animals was observed and three notable metameters i.e., hypersalivation, immobility and excessive defecation were scored in each animal by placing it individually in a box with grids (5 cm × 5 cm)^19^. Salivation was scored using the following numerical scale: 0 = Absence of response, 1 = Jaw and fur noticeably wet but no drooling noted, 2 = Maximal response with saliva actively dripping from the jaws. Immobility of the animal was scored using the following scale: 0 = Free movement of the animal, 1 = Movement of the animal restricted to 3–6 squares, 2 = Movement of the animal restricted to less than 3 squares and defecation was scored as 0 = normal, 1 = mild, 2 = severe. The sum of the scores for each metameter at regular intervals as well as a cumulative score was used to compare the effect of muscarine with that of the extract. Another set of animals (n = 3) were studied for the effect of muscarine and extract on heart and bladder in terms of heart rate and extent of micturition respectively. Each mice was placed individually on dried filter papers immersed in alkaline phenol-red and total area of urine spots was measured to indicate the extent of micturition^20^. Heart rate was measured using the blood pressure monitoring machine (Muromachi Kikai Co. Ltd).

### Pharmacokinetics and biodistribution studies of radiolabelled muscarine

#### Radio labelling of muscarine chloride (MC)

The radiolabelling studies were carried out at Institute of Nuclear Medicine and Allied Sciences (INMAS), Delhi, India. The compound muscarine chloride (MC) (0.5 mg) was radiolabelled with Technetium pertechnetate (^99m^TcO_4_^−^) at the hydroxyl groups using stannous chloride as the reducing agent^21^. Briefly, 100 μL of ^99m^TcO_4_^−^ (approximately 5 mCi, obtained by solvent extraction from Molybdenum) was mixed with stannous chloride solution (200 μg) in 10% acetic acid to reduce the Technetium. The pH was adjusted to 6.5–7.0 using 0.5 M sodium bicarbonate. To this, 0.5 mg of MC was added and incubated for 15 min at room temperature. The labelled formulation thus obtained was stored in sterile evacuated sealed vials for subsequent studies.

#### Radio labelling efficiency

Radiolabelling efficiency (LE) of technetium labelled muscarine chloride formulation was determined by ascending instant thin layer chromatography (ITLC) using silica gel (SG)-coated fibre glass sheets, approximately 10 cm in length (Gelman Science Inc., Ann Arber, MI, USA). ITLC was performed using acetone as the mobile phase. 2–3 μL of radiolabelled formulation was applied at a point of 1 cm from one end of an ITLC-SG strip. The strip was developed in acetone and solvent front was allowed to reach approximately 8 cm from the origin. The strip was cut into two equal halves and radioactivity in each segment was determined in a gamma-ray counter (Caprac Capintech Inc., NJ. USA). The free ^99m^TcO_4_^-^ moved with the solvent (R_f_ = 0.9) while radiolabelled formulation remained at the point of application. Percent labelling efficiency was calculated as follows:

$${\text{Labelling}}\,{\text{efficiency}}\,\left( \% \right)\, = \,{\text{T}}\, \times \,{1}00/{\text{T}}\, + \,{\text{B}};$$

where T is radioactive counts at top and B is the radioactive counts at bottom.

#### In vitro stability of radio labelled formulations

The in vitro stability of radio labelled muscarine chloride formulation was determined by mixing 100 μL of radio labelled formulation with 1.9 mL of human serum and incubating it at 37 °C. Any change in radio labelling efficiency was monitored at regular intervals over a period of 24 h by ITLC as described previously.

#### Pharmacokinetics and biodistribution of muscarine

The study was performed in normal, healthy, female New Zealand rabbits (n = 3) weighing 2.5–3 kg^22–24^. Animals were injected with 1.5 mCi ^99m^TcO_4_^−^ labelled muscarine formulation through the dorsal ear vein. Blood (0.2 mL) was withdrawn through the vein of another ear at periodic intervals in pre-weighed tubes. The weight of the blood withdrawn at each interval was noted and the radioactivity measured using a gamma-ray spectrometer (Caprac Capintech Inc., NJ, USA). The activity present in total blood was calculated by considering 7.3% of total body weight as total blood volume. Static scintigraphy images were acquired at periodic intervals on administration of the radiolabelled formulation by positioning the animal under the gamma camera. The results were expressed as percentage of the injected dose.

## Results and discussion

### Volatile compounds of *Inocybe virosa*

**Figure 1:**
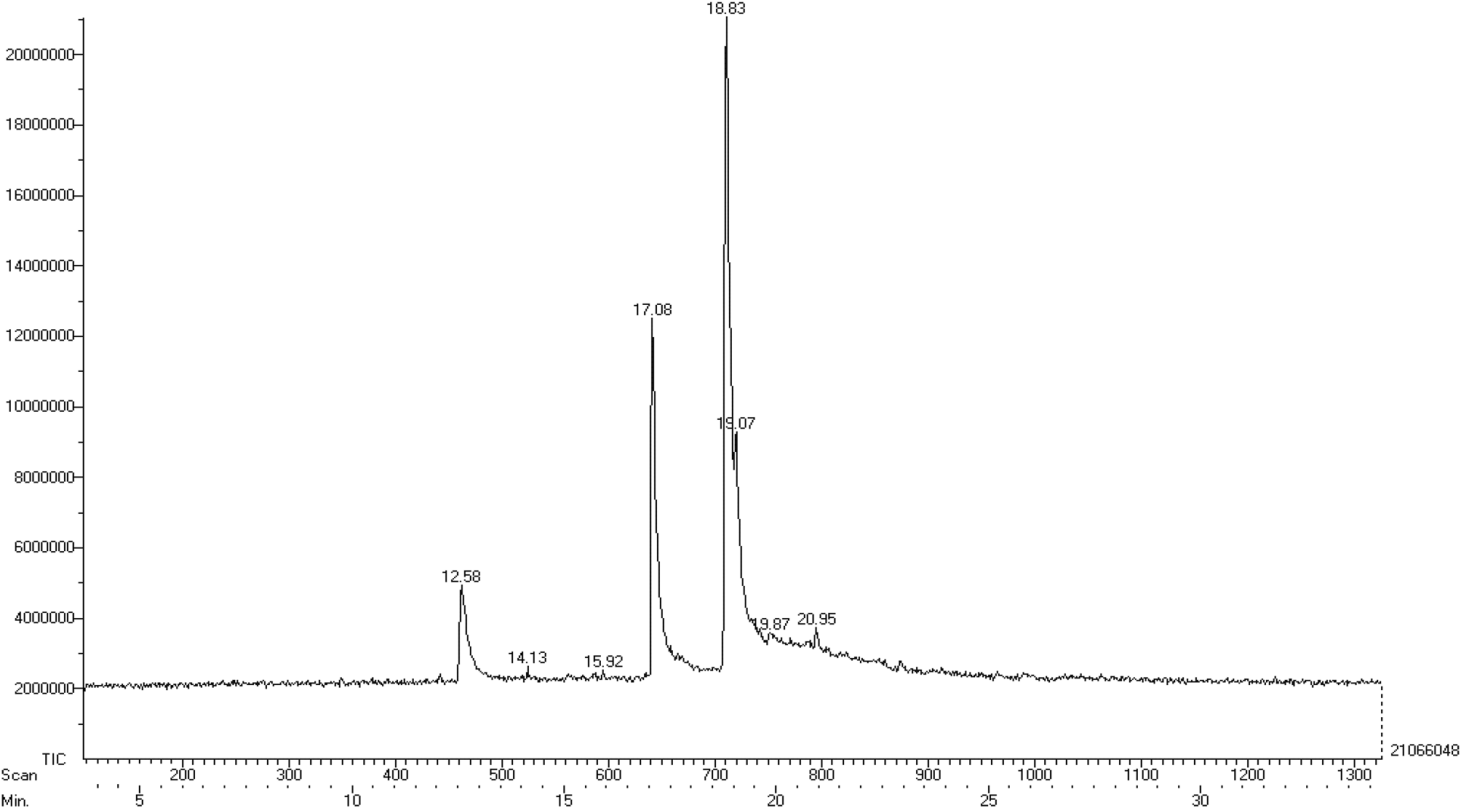
Gas chromatogram of hydro-ethanolic extract of *Inocybe virosa*: Total ion current (TIC) chromatogram from the GC separation of volatile compounds of the extract.

**Figure 2:**
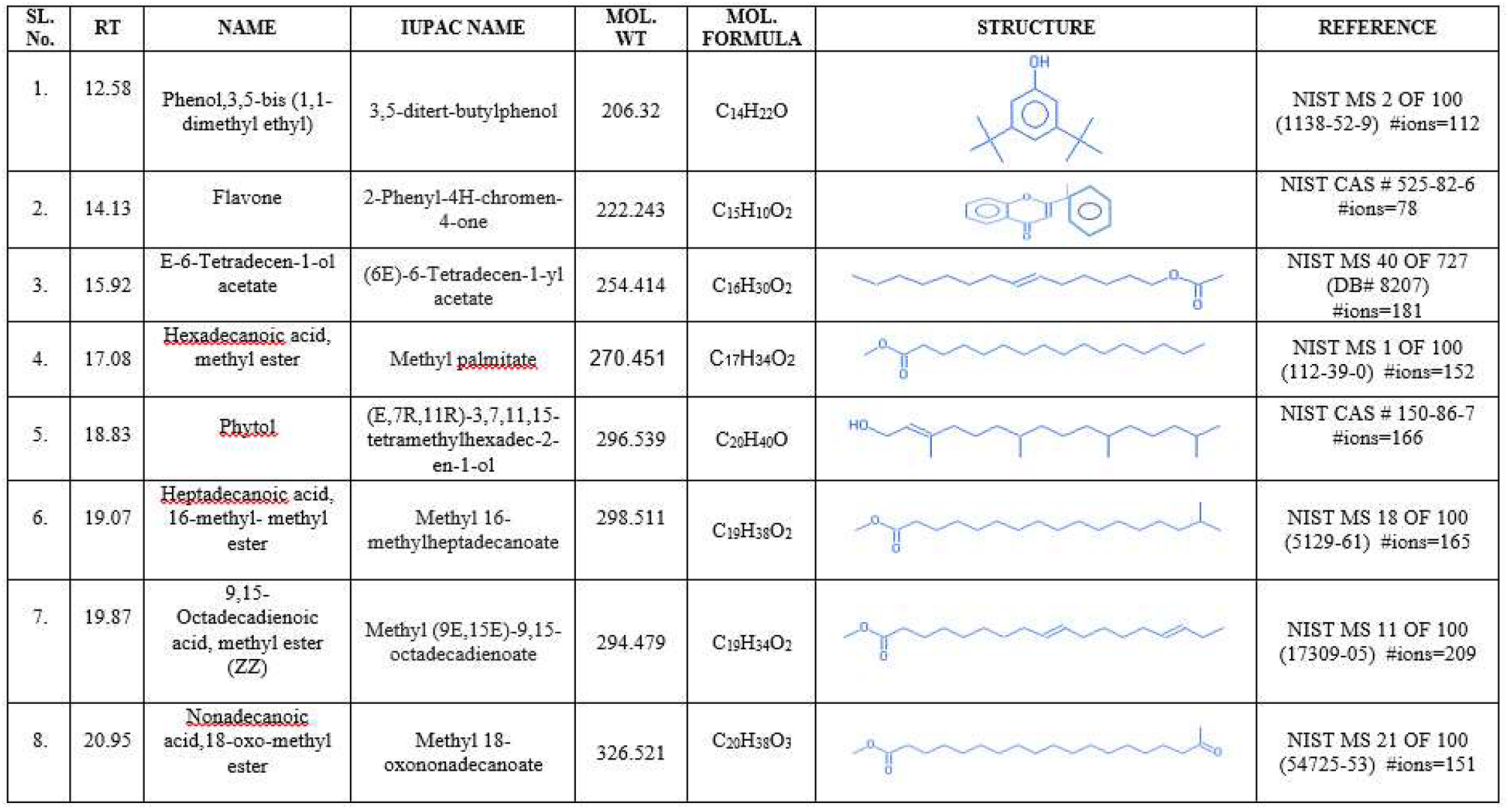
Compounds identified in hydro-ethanolic extract of Inocybe virosa by gas chromatography.

The presence of volatile toxic metabolites in *Inocybe virosa* was verified using gas chromatography. Figure 1 presents the gas chromatogram of hydro-ethanolic extract of *Inocybe virosa*. The compounds were identified on the basis of their retention times. Peak assignment was confirmed using NIST library of GC–MS. The molecular structures and molecular formulae of the compounds as shown in Fig. 2 were obtained based on the MS fragmentation pattern. The volatile profile of the extract showed a majority of fatty acid esters: E-6-tetradecen-1-ol acetate, hexadecanoic acid-methyl ester, heptadecanoic acid, 16-methyl- methyl ester, 9,15-octadecadienoic acid-methyl ester (ZZ), nonadecanoic acid-18-oxo-methyl ester. A previous study has also shown that fatty acid esters constituted a major fraction of mushrooms such as *Pleurotus eous*^25^. Among the esters identified, hexadecanoic acid, methyl ester (methyl palmitate) was reported to be associated with acaricidal or insecticidal activity^26^ and has shown to cause neurotoxicity in mites^27^. Other constituents identified in the extract were phenol, 3,5-bis(1,1-dimethyl ethyl), Phytol and Flavone. It has been reported that flavone is associated with nematicidal effects^28^. Phenolic toxicity has been demonstrated on human colonic epithelial cells^29^ and is identified as an environmental pollutant affecting aquatic macrophytes^30^. It can be speculated that these compounds may have possibly contributed to the toxicity of *Inocybe virosa*, although a more detailed study is required to ascertain this inference. It is reported that the volatile composition is independent of the mushroom state (fresh, frozen or dried)^31^ and therefore, the volatile secondary metabolites of wild mushroom species can serve as biomarkers for species discrimination as well^32,33^.

### Identification of muscarine in *Inocybe virosa*

**Figure 3:**
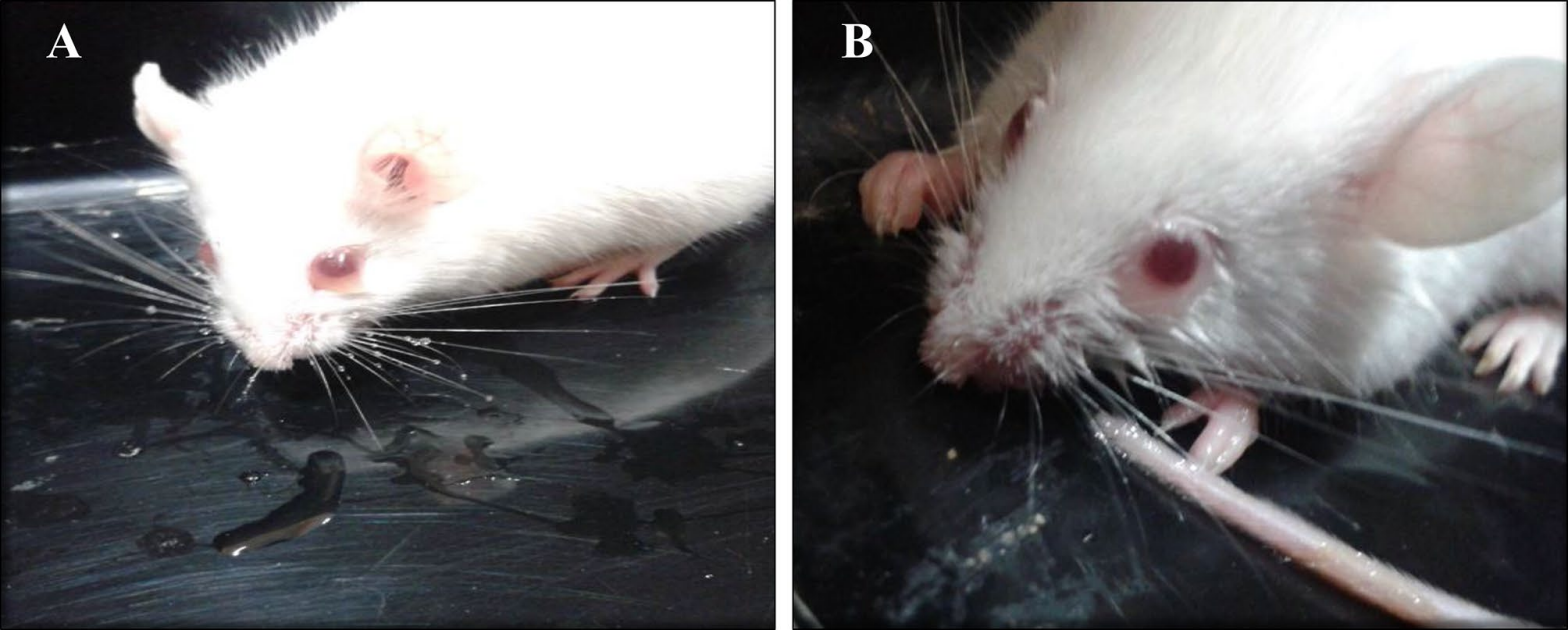
Muscarinic response observed in mice administered with *Inocybe virosa* extract (**A**) Hypersalivation (**B**) Miosis.

**Figure 4:**
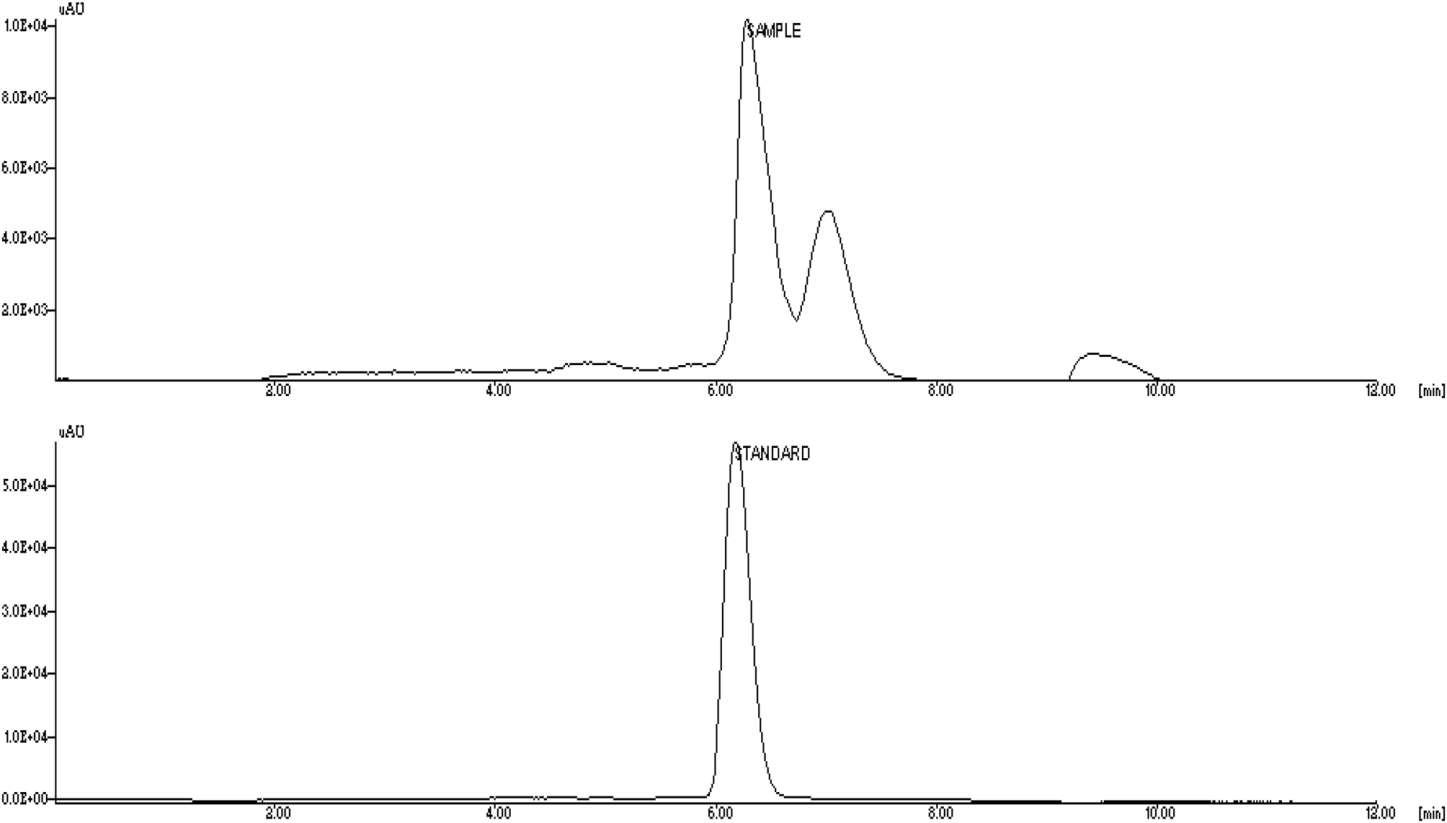
RP-HPLC analysis of *Inocybe virosa* for the presence of muscarine (**A**) mushroom extract, (**B**) muscarine standard.

**Figure 5:**
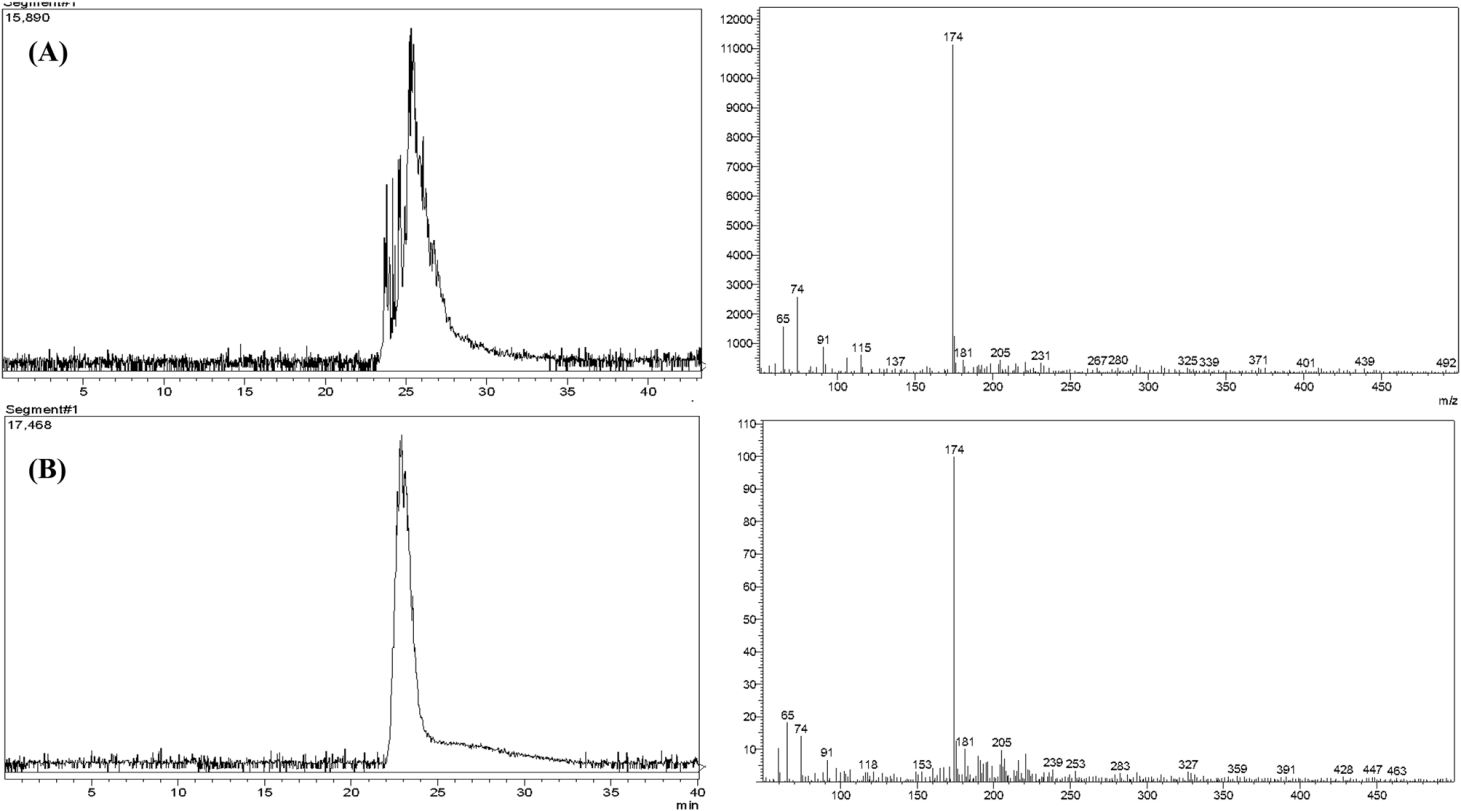
LC–MS validation for the presence of muscarine in *Inocybe virosa* extract (**A**) total ion current (TIC) chromatogram from the HPLC separation of non-volatile compounds present in the extract; Muscarine detected in *Inocybe virosa* extract by MS data based on mol wt, (**B**) total ion current (TIC) chromatogram from the HPLC separation of Muscarine standard (molecular weight—174 g/mol) with its MS data.

The symptoms of muscarinic syndrome observed in mice on administration of *Inocybe virosa* extract (Fig. 3) was an indication to verify the presence of muscarine in this mushroom. *Inocybe* species are known to contain muscarine and its detection methods have varied widely over the years. Chemotaxonomic tests^34^, paper chromatography using Thies and Reuther’s reagent^35^ and gas chromatography^11^ were used to identify muscarine in *Inocybe* species. Liquid Chromatography methods were developed and coupled to mass spectrometry for specific and rapid identification of muscarine in matrices such as food^36^ and urine^37^. The procedure given by Yoshioka et al.^16^ was found best suited for our laboratory settings. However, 0.1% (v/v) formic acid (Mobile phase A) was replaced by K_2_HPO_4_ buffer to obtain a better resolution and sharper peaks. The procedure was standardized by varying the concentration and pH of the buffer. The salt concentration was maintained at minimal levels to prevent any interference (5 mM and 10 mM), while not compromising on the resolution of chromatographic peaks. pH variations ranged from acidic to neutral (2.5, 5 and 7) as the compound of interest was cationic in nature. The combination of 5 mM at pH 2.5 was found optimum for detection of muscarine. This is one of the first reports of the occurrence of muscarine in *Inocybe virosa*. The estimated toxin content was 0.27 mg/g in the extract (Fig. 4). It was evident that hydro-ethanolic extraction increased toxin concentration approximately ten folds in comparison to the crude methanolic extraction. Our HPLC finding was further validated by LC–MS analysis based on its molecular weight (174 g/mol) (Fig. 5). Our results reiterate the point made by Kosentka et al.^38^ that the occurrence of muscarine in species of the genus *Inocybe* is an ancestral trait*.* From these results, it can be clearly inferred that the manifestation of muscarinic symptoms was an outcome of the presence of muscarine in the mushroom.

### In vitro toxicity of *Inocybe virosa*

#### Effect on cell viability

**Figure 6:**
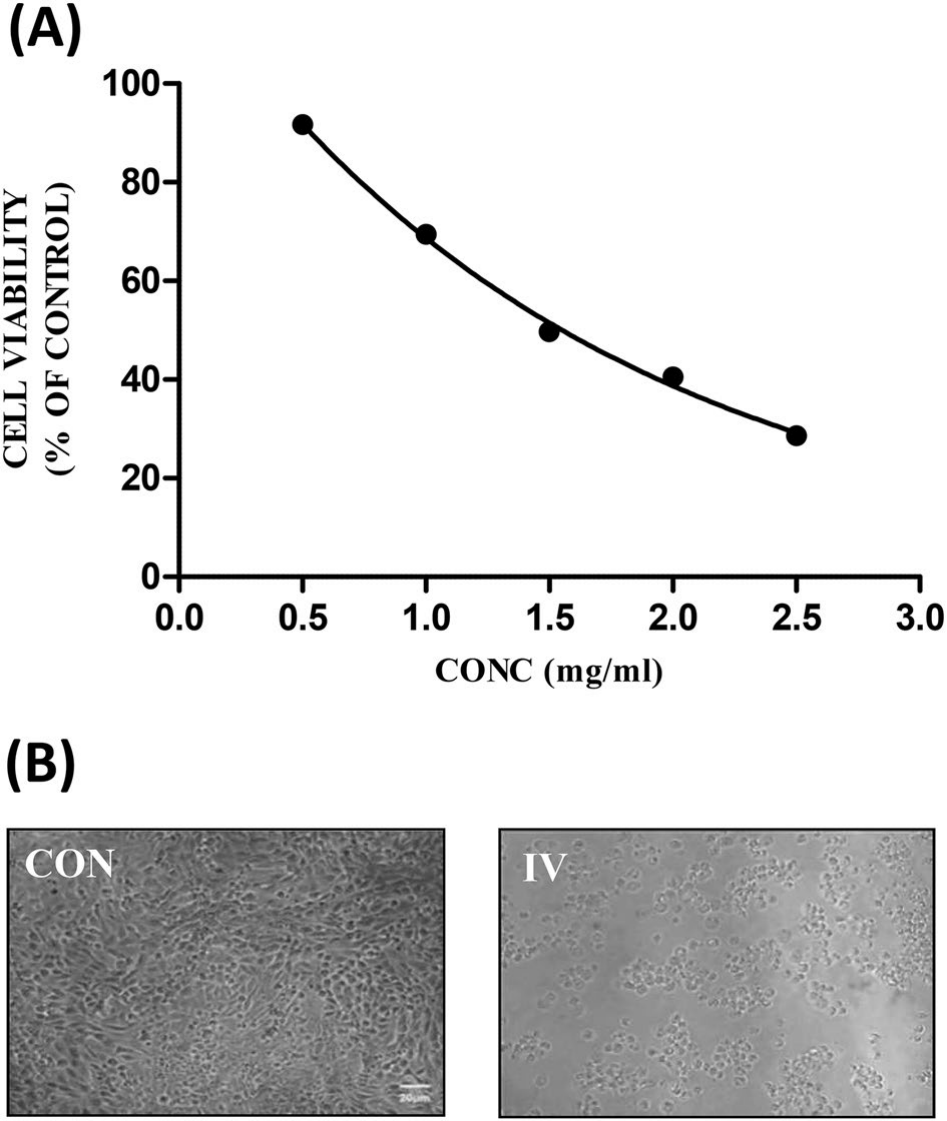
(**A**) The dose–response effect of IV extract on NCM460 cells; IC_50_ value—0.63 mg/mL, (**B**) the effect of IV extract on the morphology of NCM460 cells under phase contrast microscope (20 ×).

**Figure 7:**
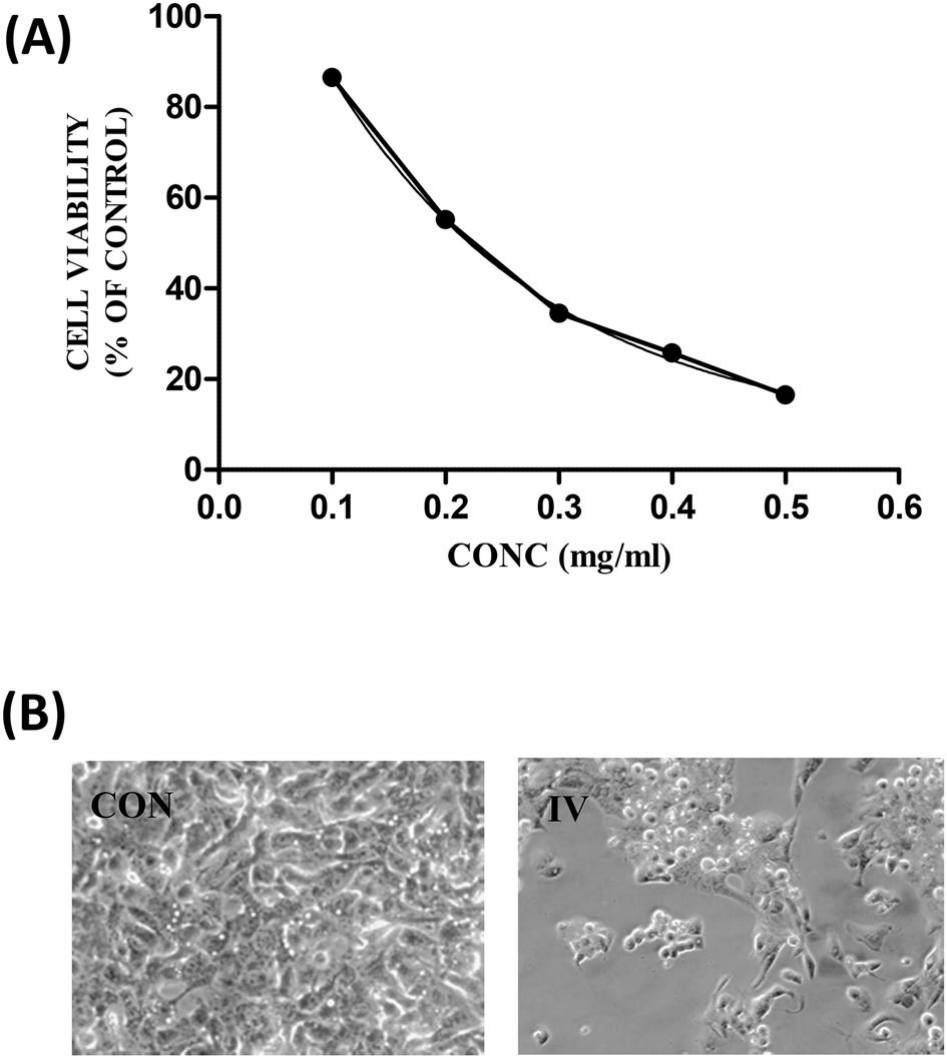
(**A**) The dose–response effect of IV extract on Chang liver cells; IC_50_ value—0.15 mg/mL, (**B**) the effect of IV extract on the morphology of Chang liver cells under phase contrast microscope (40 ×).

On 24 h exposure to the mushroom extract, there were distinct cytotoxic effects on the morphological structure of the NCM460 intestinal cells (Fig. 6). The cell viability showed a significant (*p* < 0.05) reduction in case of the mushroom extract with respect to control. Similarly, in case of the Chang liver cells, a dose-dependent decrease in cell viability was observed on exposure to different concentrations of the extract (Fig. 7) as it was noted in the intestinal cells. *Inocybe virosa* was toxic to hepatocytes with a low IC_50_ value (0.18 mg/mL).

For the preliminary in vitro study, NCM460 colon epithelial cell line was selected to represent the intestinal barrier as these cells are the earliest to come in contact with the mushrooms on their consumption. The toxic potential of the mushroom was assessed considering their effects on cell morphology and cell viability. Morphological changes and abnormalities such as cell shrinkage and distortion in cell shape were evident in the cells exposed to the extract as shown in Fig. 6. *Inocybe virosa* had toxic effect on intestinal cells showing a significantly low IC_50_ value of 0.63 mg/mL. This indicated a marked possibility of intestinal toxicity associated with their consumption. Our finding of intestinal toxicity associated with *Inocybe virosa* stands in agreement with the severe gastrointestinal symptoms documented on consuming this mushroom^39–41^.

Liver being an organ of defense against the exogenous compounds, an in vitro toxicity evaluation using the representative Chang liver cell line was considered in the study. Cell viability was monitored to assess the overall effect on the cells. As evident from Fig. 7, the mushroom extract exhibited potential hepatotoxicity. A previous study by Kawaji et al.^42^ demonstrated in vitro hepatotoxicity on exposure to toxic *Amanita* species such as *Amanita abrupta*, *Amanita virosa*, and *Amanita volvata*. This is also one of the first reports of in vitro toxicity in liver cell lines w.r.t *Inocybe virosa*.

### In vivo toxic effects of *Inocybe virosa*

#### General observations

On oral administration of the mushroom extract at the two selected doses in acute toxicity evaluation, the animals showed no mortality during the study period. Also, the administration of extract caused no significant reduction in body weight of the animals in comparison to control. However, muscarinic symptoms viz., excessive salivation, lacrimation, and urination were markedly observed in a dose-dependent manner.

#### Acute toxic effects

*Inocybe virosa* at 500 mg/kg bwt elicited the highest WBC count (9.13 ± 1.93 10^3^/µL). However, on day 14, the WBC count was normal as shown in Table 1. The other haematological parameters such as PLTs, RBCs, Hgb, and Hct were not significantly different from the control values. A significant increase was noted in KFTs and LFTs after 24 h in case of *Inocybe virosa* (Tables 2 and 4). The levels of the liver enzymes though slightly lowered by day 14, they were significantly higher in case of *Inocybe virosa* at 500 mg/kg bwt (Table 3).

**Table 1:** Acute toxicity study: effect of the mushroom extracts on hematological parameters.

Parameters	24 h exposure			14 day exposure		
	Control	Inocybe virosa extract (mg/kg bwt)		Control	Inocybe virosa extract (mg/kg bwt)	
		250	500		250	500
WBC (10^3^/µL)	4.06 ± 1.02	7.96 ± 1.55*	9.13 ± 1.93*	3.94 ± 1.50	3.42 ± 0.45	4.18 ± 0.42
PLT (10^5^/µL)	994.27 ± 236.63	723.66 ± 138.22	1,182.3 ± 184.21	1,012.4 ± 175.89	1,028.0 ± 184.54	1,117.0 ± 270.21
RBC (10^6^/µL)	8.84 ± 1.18	8.24 ± 1.42	8.03 ± 0.26	9.23 ± 0.48	9.41 ± 1.15	9.50 ± 0.48
Hgb (g/dL)	13.72 ± 1.27	11.63 ± 3.49	12.50 ± 1.29	14.05 ± 0.59	13.36 ± 1.44	13.24 ± 0.22
Hct (%)	42.54 ± 3.36	42.40 ± 6.16	45.80 ± 0.88	46.23 ± 4.14	45.06 ± 6.25	48.50 ± 3.04

*represents significant difference with respect to control at *p* < 0.05.

**Table 2:** Acute toxicity study: effect of the mushroom extracts on kidney functioning parameters.

Parameters	24 h Exposure			14 day exposure		
	Control	Inocybe virosa extract (mg/kg bwt)		Control	Inocybe virosa extract (mg/kg bwt)	
		250	500		250	500
CRE (mg/dL)	0.27 ± 0.06	0.44 ± 0.06*	0.59 ± 0.07*	0.24 ± 0.03	0.31 ± 0.07	0.33 ± 0.06
BUN (mg/dL)	23.31 ± 3.50	32.65 ± 2.49*	34.59 ± 3.42*	20.79 ± 3.68	23.55 ± 0.90	30.73 ± 3.18*
TPR (mg/dL)	5.03 ± 0.26	6.99 ± 0.13*	7.03 ± 0.05*	4.19 ± 0.58	4.98 ± 0.92	5.60 ± 1.45

*represents significant difference with respect to control at *p* < 0.05.

**Table 3:** Acute toxicity study: effect of the mushroom extracts on liver functioning parameters.

Parameters	24 h exposure			Control	14 day exposure	
	Control	Inocybe virosa extract (mg/kg bwt)			Inocybe virosa extract (mg/kg bwt)	
		250	500		250	500
AST (IU/L)	69.29 ± 8.11	95.55 ± 2.53*	140.82 ± 6.04*	57.07 ± 9.73	71.03 ± 7.09	83.49 ± 3.91*
ALT (IU/L)	81.25 ± 16.65	120.33 ± 1.89*	159.35 ± 2.52*	69.81 ± 10.32	83.26 ± 6.73	115.82 ± 10.16*
ALK (IU/L)	127.37 ± 8.63	185.63 ± 7.57*	281.36 ± 6.28*	131.95 ± 9.80	133.35 ± 9.67	174.05 ± 3.98*
TBIL (mg/dL)	0.37 ± 0.03	0.49 ± 0.07*	0.96 ± 0.06*	0.28 ± 0.05	0.31 ± 0.02	0.44 ± 0.03*

*represents significant difference with respect to control at *p* < 0.05.

**Figure 8:**
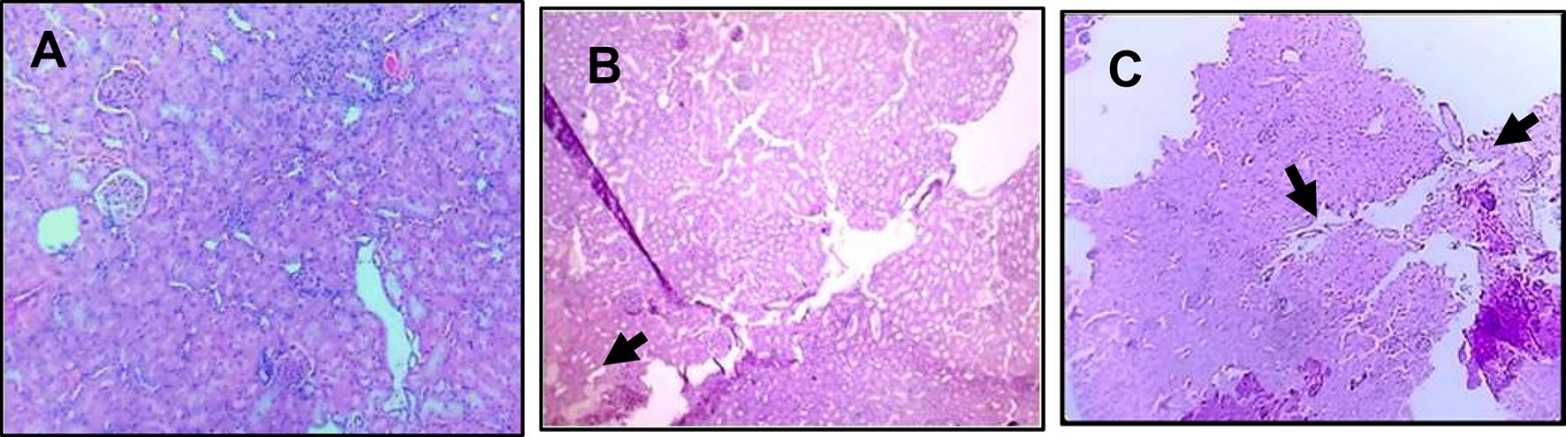
Acute toxicity study: Effect of mushroom extracts at 250 and 500 mg/kg bwt on kidney histology after 24 h exposure (**A**) control, (**B**) IV 250 showing mild damage, (**C**) IV 500 showing glomerular and tubular distortion with foci of haemorrhage.

**Figure 9:**
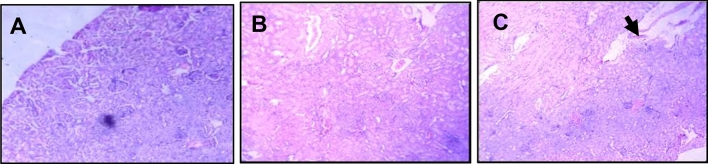
Acute toxicity study: effect of mushroom extracts at 250 and 500 mg/kg bwt on kidney histology on 14 days exposure (**A**) control, (**B**) IV 250, (**C**) IV 500 showing mild tubular distortion.

**Figure 10:**
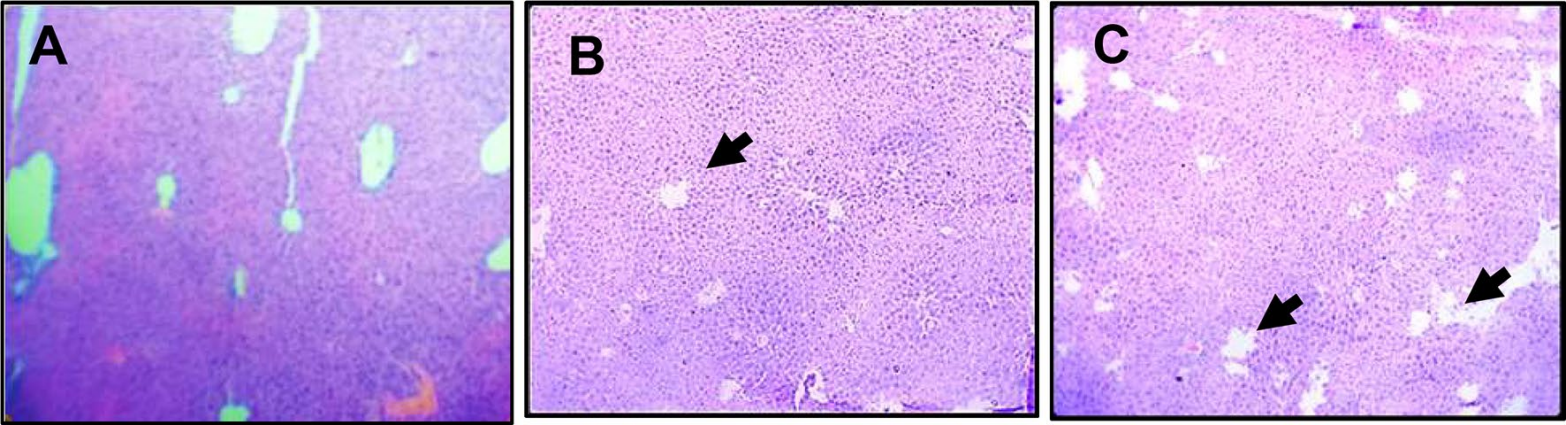
Acute toxicity study: Effect of mushroom extracts at 250 and 500 mg/kg bwt on liver histology after 24 h exposure (**A**) control (**B**) IV 250 showing mild feathery degeneration; (**C**) IV 500 showing showing hepatic lobular distortion.

**Figure 11:**
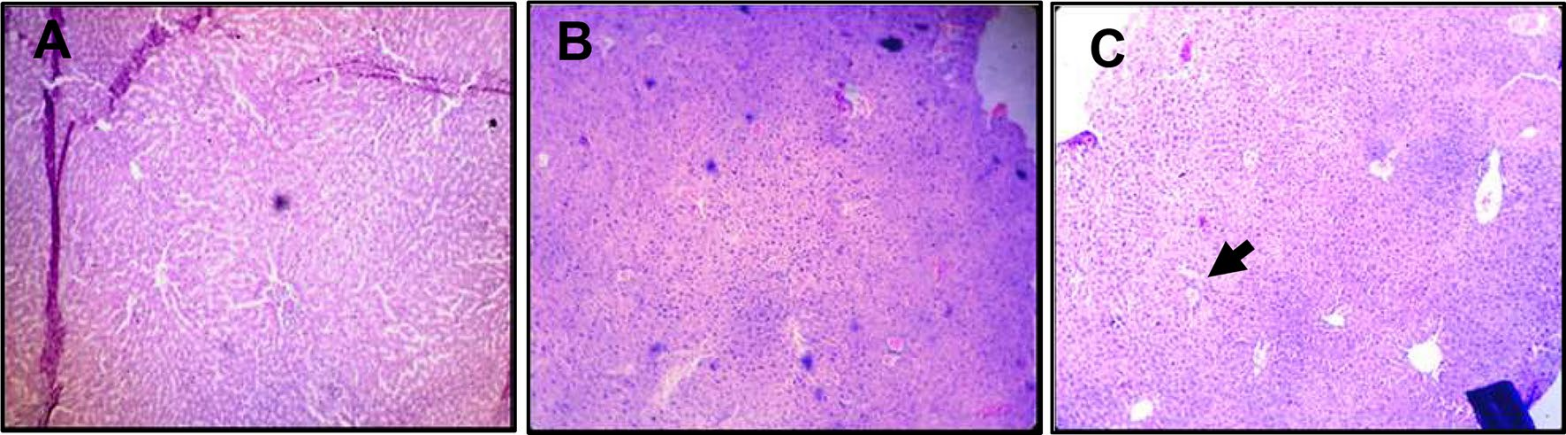
Acute toxicity study: Effect of mushroom extracts at 250 and 500 mg/kg bwt on liver histology on 14 days exposure (**A**) control, (**B**) IV 250, (**C**) IV 500 showing mild feathery degeneration.

**Figure 12:**
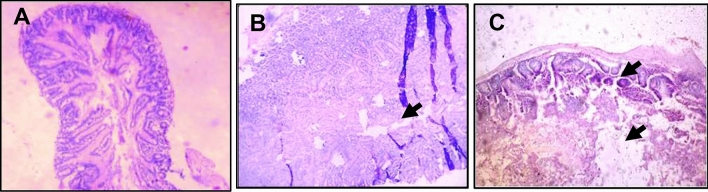
Acute toxicity study: effect of mushroom extracts at 250 and 500 mg/kg bwt on intestine histology after 24 h exposure (**A**) control, (**B**) IV 250 showing mild hyperplasia, (**C**) IV 500 showing sloughing of mucosa, distortion of glands and non-specific inflammation.

**Figure 13:**
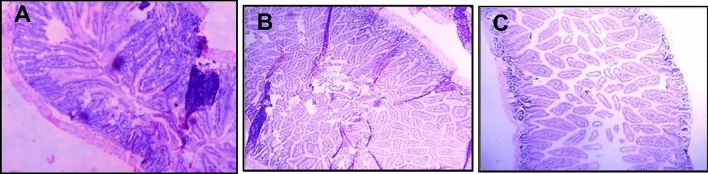
Acute toxicity study: effect of mushroom extracts at 250 and 500 mg/kg bwt on intestine histology on 14 days exposure (**A**) control, (**B**) IV 250, (**C**) IV 500. No pathology observed.

There were degenerative changes in kidney at both doses after 24 h of administration of *Inocybe virosa*. At 500 mg/kg bwt, glomerular and tubular distortion with foci of haemorrhage was seen while the damage was milder at 250 mg/kg bwt. The tissue distortion at higher dose of *Inocybe virosa* was observed on day 14 also (Figs. 8 and 9). With respect to liver histology, *Inocybe virosa,* at both the doses caused hepatic lobular distortion after 24 h and a mild feathery degeneration was observed on day 14 at higher dose only (Figs. 10 and 11). *Inocybe virosa* extract showed mild hyperplasia at the lower dose while at 500 mg/kg bwt, sloughing of mucosa with non-specific inflammation and distortion of glands w.r.t intestinal tissue after 24 h. There was no intestinal pathology noted on day 14 (Figs. 12 and 13). The brain histology however showed no obvious pathology.

In vivo acute toxicity study was useful in verifying if an oral consumption of the selected mushroom *Inocybe virosa* caused any in vivo systemic toxic effects and also to identify if there was any specific target organ. Female mice were preferred for the study as they are generally more sensitive to toxic effects^43^. As the most significant human exposure to mushrooms occurs through ingestion, oral route was chosen for this study. The yield on extraction was about 3.5% of fresh weight of the mushrooms and therefore a dose of 500 mg mushroom extract/kg body weight (bwt) for mice (25–30 g) when extrapolated to humans (60 kg) is approximately equivalent to one serving of mushroom per meal with conversion factor of 1/12^44^. The OECD guideline TG 423 (2001) for acute toxicity was followed but at doses of the mushroom extract which had relevance to daily intake of mushrooms by humans. The onset of symptoms on mushroom poisoning is usually within 6-24 h^45^. However, the clinical manifestations in many poisonings may also have a latency period up to 14 days^46^. Therefore in our study, 24 h and 14 days were the time points considered to evaluate toxic effects in response to a single oral administration of the mushroom. Considering the hematological parameters, increased level of WBCs (Table 1) indicated an inflammatory response. A report of leucocytosis (an increase in WBC count) on account of wild mushroom poisoning in Iran^47^ supports our finding. In addition to hematological parameters, clinical biochemistry data also plays an important role in determining toxic effects. As mushroom poisonings are generally associated with gastrointestinal, renal, hepatic, or neurological effects^48^, small and large intestines, kidney, liver and brain were examined for pathological signs.

ALT and AST present in cytoplasm of hepatocytes are sensitive markers of liver tissue damage, as they show a marked elevation in their activities in the bloodstream on account of any liver injury^49^. ALP which is also particularly concentrated in liver shows an increase in case of distortion in hepatic architecture. The biochemical parameters ALP, ALT, AST including total bilirubin are therefore reliable indicators of hepatic functions; while total protein, creatinine, and urea levels are good measures of renal functions^50^. The mushroom *Inocybe virosa*, increased levels of ALT, AST, ALP and TBIL after 24 h (Table 3) indicating altered functional status of liver with hyperbilirubinemia which correlated well with histopathological observations made in liver tissue (Fig. 10 and 11). It is thus reasonable to speculate that increased activity of these enzymes could be due to hepatic damage and subsequent leakage of these enzymes into circulation. These sensitive biomarkers were elevated in accidental poisonings in humans with wild mushrooms^51,52^ and also in case of amanitin intoxication^53^. This clinical data stands in agreement with our findings. The persistence of this damage up to day 14 in case of *Inocybe virosa* is of toxicological significance and strongly affirms its hepatotoxicity. There are reports of acute hepatitis on consumption of wild mushrooms^54^ which substantiates the hepatotoxicity noted in our study. Pathology was noted in kidney tissue on exposure to *Inocybe virosa* at both doses after 24 h and at the higher dose on day 14 (Figs. 8 and 9). This was in consonance with the significant increase shown in KFTs (Table 2) indicating that kidney was also a target organ of this mushroom.

The evident toxic effects of *Inocybe virosa* well justify its nomenclature *virosa* (meaning full of poison)^55^.The typical muscarinic symptoms on the administration of *Inocybe virosa* speculates the presence of toxin muscarine in this new and unexplored *Inocybe* species. Literature indicates that the consumption of *Inocyb*e species is associated with muscarinic syndrome^56^. Muscarine is a characteristic component of genus *Inocybe,* and in fact, our study affirms the presence of muscarine in *Inocybaceae* family as an ancestral trait^38^.

### Effect of in vitro digestion on muscarine

**Figure 14:**
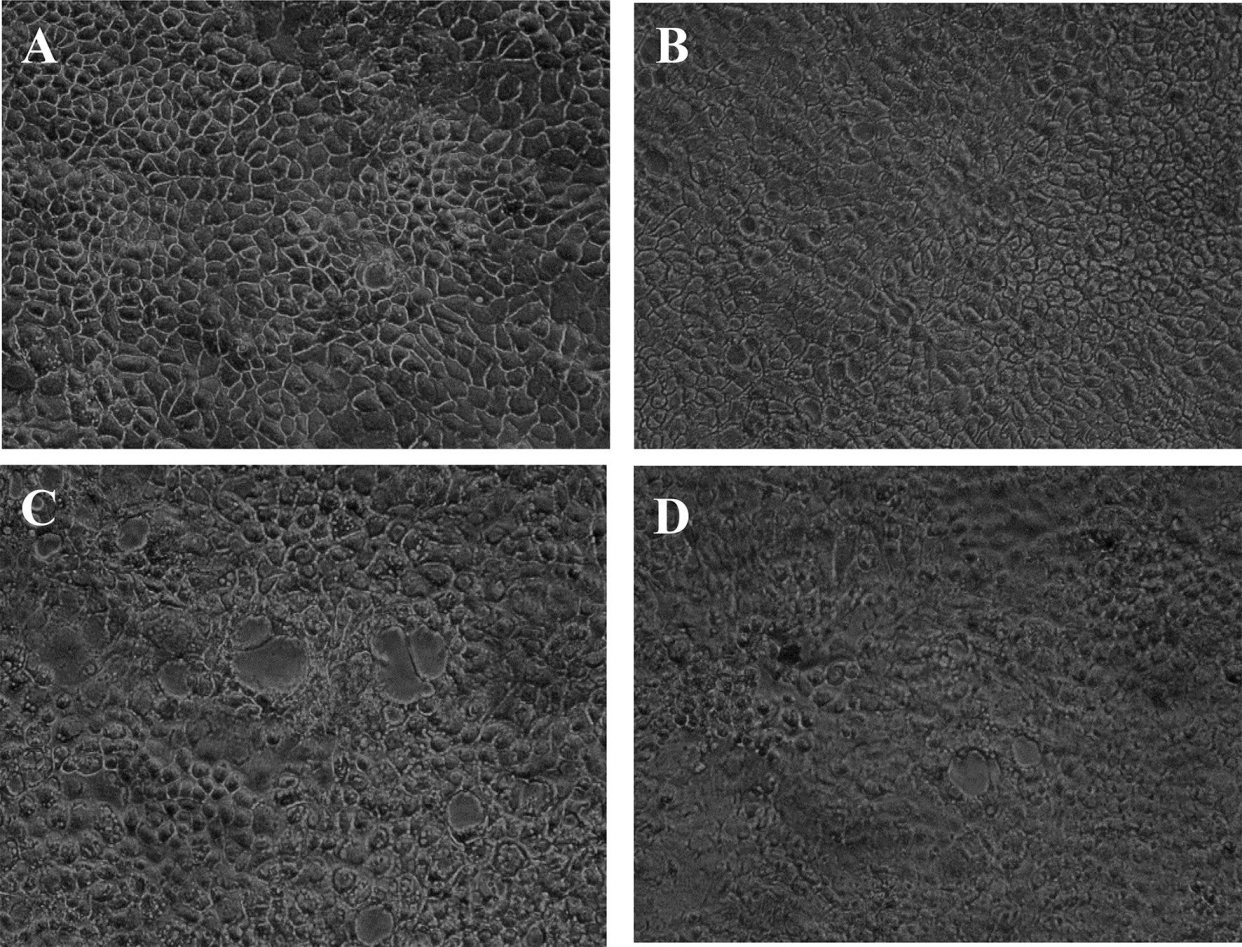
Cytotoxicity of the digestate: Effect on cell morphology (**A**) control, (**B**) digestate control, (**C**) digestate, (**D**) extract.

**Figure 15:**
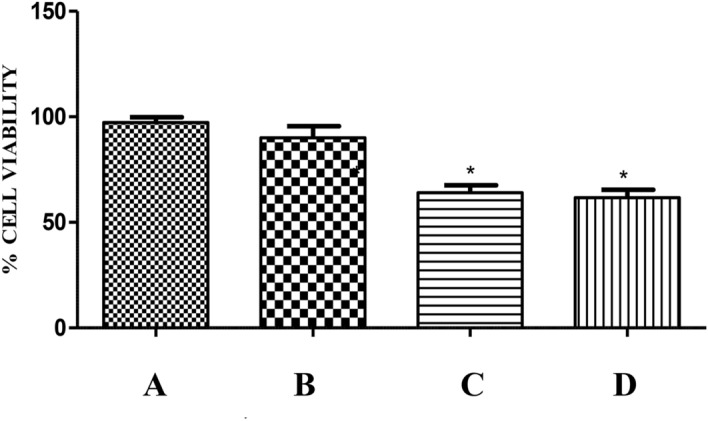
Cytotoxicity of the digestate: Effect on cell viability (**A**) control, (**B**) digestate control, (**C**) digestate, (**D**) extract. * represents significant difference w.r.t. Control *p* < 0.05.

**Figure 16:**
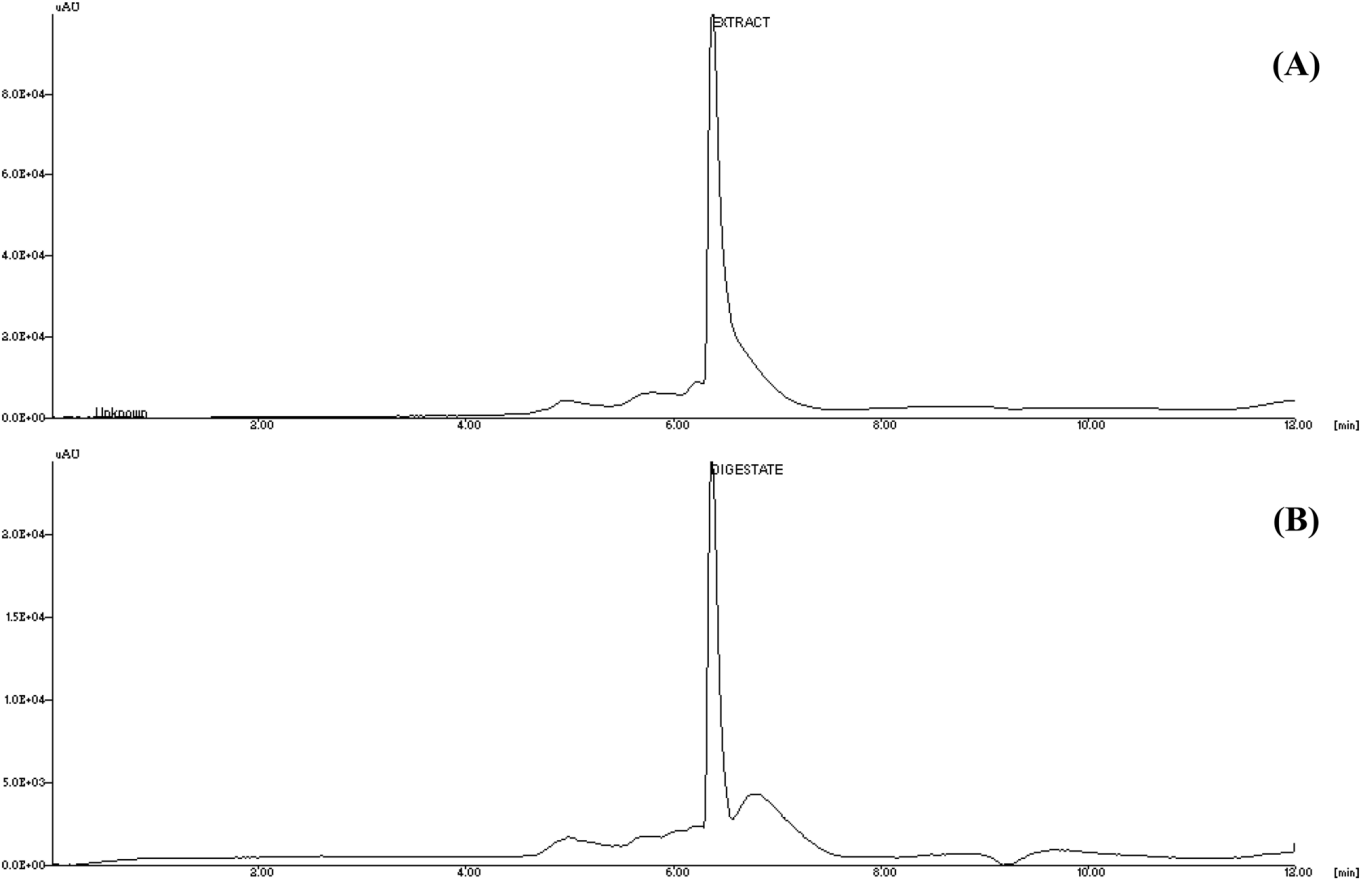
Effect of in vitro digestion on the muscarine content in *Inocybe virosa* (**A**) extract before digestion showing 2.92 mg/g, (**B**) extract after digestion showing 2.61 mg/g.

The present study also attempted to substantiate the toxic effects associated with the oral intake of the mushroom (usually as a part of diet) using an in vitro model. Therefore, the effect of digestive enzymes and the associated pH variations on muscarine content and toxicity of the extract was analysed by simulating the digestion conditions in mouth, stomach and small intestine. The effect of the digestate was studied on Caco-2 intestinal cells which were representative of the intestinal barrier^57^. The digestate represented the bioaccessible fraction which is most likely to get absorbed by the intestinal enterocytes. Its effects were assessed based on the morphological changes observed in cells exposed to it (Fig. 14) and estimated in terms of cell viability (Fig. 15). There was no significant decrease in cell viability with respect to control on exposure to the digestate control (the mixture of digestive enzymes used for in vitro digestion). Hence, it can be concluded that the digestive enzymes had negligible adverse effects on the cells. In comparison to control, a significant decrease in cell viability was noted in case of digestate and extract. However, the extent of damage on exposure to extract was not significantly different from that of digestate indicating that digestion had no altering effect on the toxicity of extract. Interestingly, there was only a slight difference in the toxin content prior to (2.92 mg/g) and after (2.62 mg/g) the digestion process as shown in Fig. 16. This confirms the findings of Clark and Smith^58^ that the toxin muscarine is resistant to cooking or any other means of processing. The stability of muscarine to boiling, varying pH or the action of pepsin as pointed out by Fraser, 1957^59^ was also validated by this study.

### Analysis of muscarinic response in mice

There are evidences which indicate that the consumption of *Inocybe* species is often linked to manifestations of the muscarinic syndrome^58,60,61^. Salivation, lacrimation, urination, diaphoresis, gastrointestinal effects and emesis are some of the symptoms of muscarinic poisoning, accompanied by miosis, bronchoconstriction and bradycardia at times^62^. Malone and Brady^63^ had attempted to determine the relative muscarinic potency of *Inocybe* species based on these symptoms. The *Inocybe* mushrooms were divided into four groups based on the severity of their toxic effects^64^ (i) Species with pronounced muscarinic effect (Ex: *I. fastigiata*), (ii) Species with weak muscarinic effect (Ex: *I. umnbrina*), (iii) Species that produce an unstable effect (Ex: *I. jurans*) and (iv) Species without any muscarinic effect (Ex: *I. bongardii*). Therefore, an in vivo study is required to assess the muscarinic potency of *Inocybe virosa*. The extract was administered at a dose which had relevance to daily intake of mushrooms by humans. A dose of 500 mg mushroom extract per kg bwt for mice when extrapolated to humans (60 kg) is approximately equivalent to one serving of mushroom per meal (Conversion factor of 1/12)^44^.It was also our interest to compare the muscarinic response in mice on exposure to the extract and the equivalent muscarine content (as estimated by HPLC) in order to assess the role of muscarine in the mushroom toxicity.

**Figure 17:**
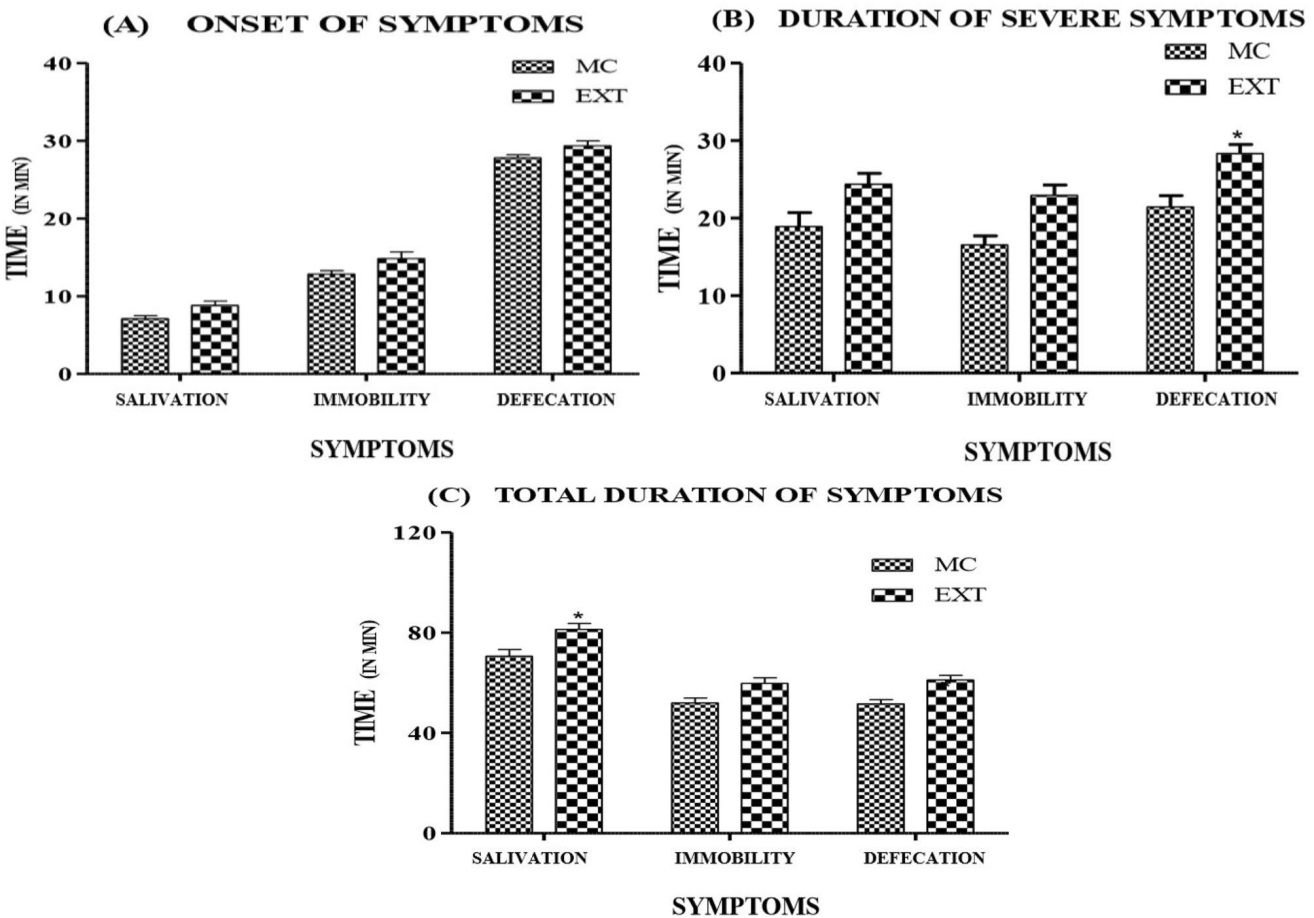
Comparison of the muscarinic response (symptoms) on administration of muscarine and *Inocybe virosa* extract (**A**) onset of symptoms, (**B**) time over which symptoms were severe, (**C**) total duration of the symptoms.

The observable symptoms were hypersalivation and miosis as shown in Figs. 3A,B respectively, which appeared within 5–10 min of administering both the extract and muscarine. This clearly pointed out that muscarine is a fast acting toxin as was suggested by de Oliveira^48^. The animals also showed excessive defecation and urination with marked phases of immobility, occasionally accompanied by myoclonic seizures. The bioassay conducted by Malone et al.^19^ in rat involved the measurement of tear secretion and an estimation of drooling. In this study, salivation, immobility and defecation were the selected metameters which were scored to assess the muscarinic response and the animals were observed till they returned to normalcy. The results showed that the extract had a slightly stronger muscarinic effect in comparison to an equivalent dose of muscarine considering total duration of the symptoms and also the time over which symptoms were severe (Fig. 16). However, the onset of symptoms was relatively earlier for muscarine than the extract, though not significantly different (Fig. 17). It was noted that salivation continued for significantly longer duration and the duration of severity for defecation was significantly higher for the extract in comparison to muscarine.

Muscarine is a non-selective acetylcholine receptor agonist. However, its cationic nature prevents its diffusion through the lipid barrier between the bloodstream and brain. Therefore, muscarine given in diet or injected into bloodstream has negligible effect on brain and does not affect the central nervous system^65^. The structural elements of muscarine closely resemble the active conformation of acetylcholine. Thus an efficient binding of muscarine to the acetylcholine receptors is favoured which causes profound activation of peripheral parasympathetic nervous system. Muscarine arriving at the synapse by diffusion from the blood binds reversibly to these receptors and turns them on and this receptor bound muscarine is not removed by acetylcholine esterase. This activation continues until the muscarine concentration falls by diffusion to a level much below the threshold and propagation of the signal is eventually terminated. Its effects are more pronounced on the M2 and M3 muscarinic acetylcholine receptors distributed on the smooth muscles and exocrine glands. Hence, muscarinic action potentiates secretions of the salivary, lacrimal and sweat glands causing characteristic symptoms viz., salivation, lacrimation and perspiration.

**Figure 18:**
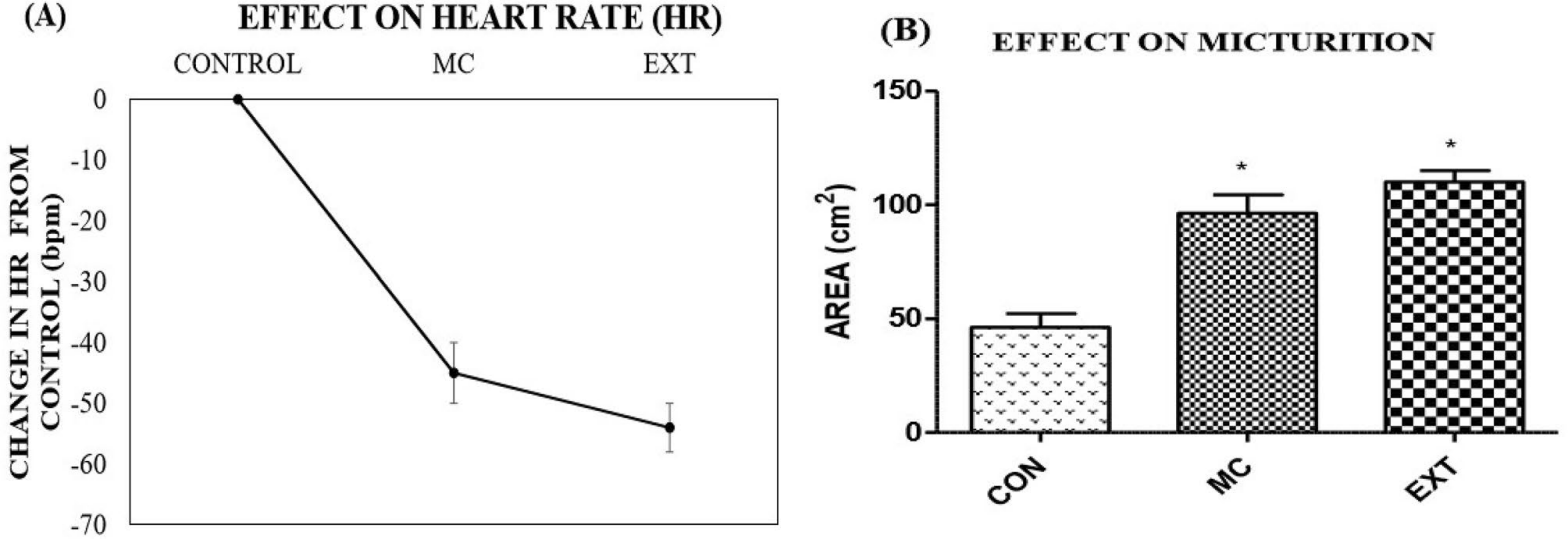
Muscarinic effect on heart and urinary bladder MC—muscarine standard, EXT—*Inocybe virosa* extract, CON—Control (**A**) heart rate; (**B**) extent of micturition.

The present study validates the findings of Lurie et al.^66^ that the poisonings by *Inocybe* species is associated with constriction of pupils, muscle spasms, diarrhoea, slowing of heartbeat and a drop in blood pressure. In a study carried out by Ochillo et al.^67^, it was demonstrated that muscarine elicited a contraction of ileal longitudinal muscles. This could possibly explain the increase in defecation (Fig. 17) as a consequence of increased persistalsis. The muscarinic effect is also exerted on smooth muscles of heart and urinary bladder. The heart rate and extent of micturition were therefore measured which were indirect indices to assess this muscarinic effect (Fig. 18). According to the toxicological studies demonstrated by Clark and Smith^58^, *Clitocybe illudens* and *Inocybe infida* caused the characteristic muscarinic effect of decreased heart rate in frogs and turtles. This finding stands in agreement to our observation of a drop in heart rate recorded in mice (Fig. 18). Muscarine syndrome can be fatal in case of ingesting large quantities of the causative species as profound activation of peripheral parasympathetic nervous system may result in convulsions and finally death^58^. The extract showed a slightly stronger muscarinic effect in comparison to the equivalent dose of muscarine. This could possibly be due to the presence of other potentiating factors like choline which contribute to the total muscarinic effect of many *Inocybe* mushrooms^68^. However, the earlier onset of the symptoms in case of muscarine as compared to the extract could be due to the negative matrix effect. This interference by other compounds is usually a common hindrance in case of biological samples^69^.

### Pharmacokinetics and biodistribution of muscarine

Muscarine was identified as one of the major toxins in *Inocybe virosa* and this was also evident from the results of the bioassay. Therefore, in order to deepen our understanding about its behaviour and biological fate in an in vivo system, pharmacokinetics was carried out. Gamma scintigraphy provides a clear picture of the distribution of a compound in a living system and proves advantageous in examining such processes non-invasively in real time^70^.Technetium-99m (^99m^Tc), the radiotracer used in the present study, is the chemical form of sodium pertechnetate obtained from molybdenum. ^99m^Tc has a half-life (t_1/2_) as short as 6 h. It emits monochromatic gamma radiations of 140 keV and one of the most widely used radiotracers in Nuclear Medicine. It exhibits a unique chemistry of forming a complex with a wide range of compounds. These properties make it an ideal choice for many applications in diagnostic, pharmaceutical and biological fields^71,72^. However, Pertechnetate ion (TcO^−4^) with an oxidation state of + 7, is chemically non-reactive and cannot complex with compounds of interest upon direct addition. Therefore, it has to be reduced to a much lower oxidation state to enable efficient labelling. Stannous chloride (SnCl_2_) is one of the commonly used reducing agent for ^99m^Tc. However, Sn^+2^ ions tend to get hydrolyzed in aqueous solution of pH 6–7 and results in formation of insoluble colloids, which also have an affinity towards ^99m^Tc. To overcome this hindrance in labelling, an acid is added which inhibits hydrolysis of Sn^+2^ ion^73^. Labelling efficiency of ^99m^Tc-muscarine chloride complex was assessed by ITLC and the results (Table 4) indicated that only about 8% of the radioactive complex dissociated even after 24 h, suggesting a very good radiolabelling efficiency. Further, Tc-99m labelled muscarine chloride was incubated with human serum and 0.9% saline at 37 °C for 24 h and it was found to be a stable complex which ascertains its suitability for in vivo application (Table 5). Rabbits were injected with a calculated amount of dose intravenously and this facilitated the study of the biodistribution of ^99m^Tc labelled muscarine chloride.

**Table 4:** Effect of incubation time on the labelling efficiency of MC with ^99m^Tc.

Incubation time (min)	% labeling efficiency
5	99.3 ± 0.6
15	98.3 ± 0.8
30	98.1 ± 0.3
60	98.6 ± 0.5
120	97.5 ± 0.3
180	96.3 ± 0.5
240	95.1 ± 0.5
1,440	92.4 ± 0.3

**Table 5:** Stability of labeled MC in human serum and physiological saline at 37 °C.

Incubation time (h)	% MC Raiolabeled	
	In human serum	In 0.9% saline
0.5	98.1 ± 0.9	99.36 ± 1.8
1	97.2 ± 1.8	98.27 ± 1.0
2	96.3 ± 1.0	99.00 ± 1.6
3	95.6 ± 1.5	98.40 ± 0.81
4	94.5 ± 1.3	97.72 ± 0.36
24	94.3 ± 1.0	97.54 ± 1.2

**Figure 19:**
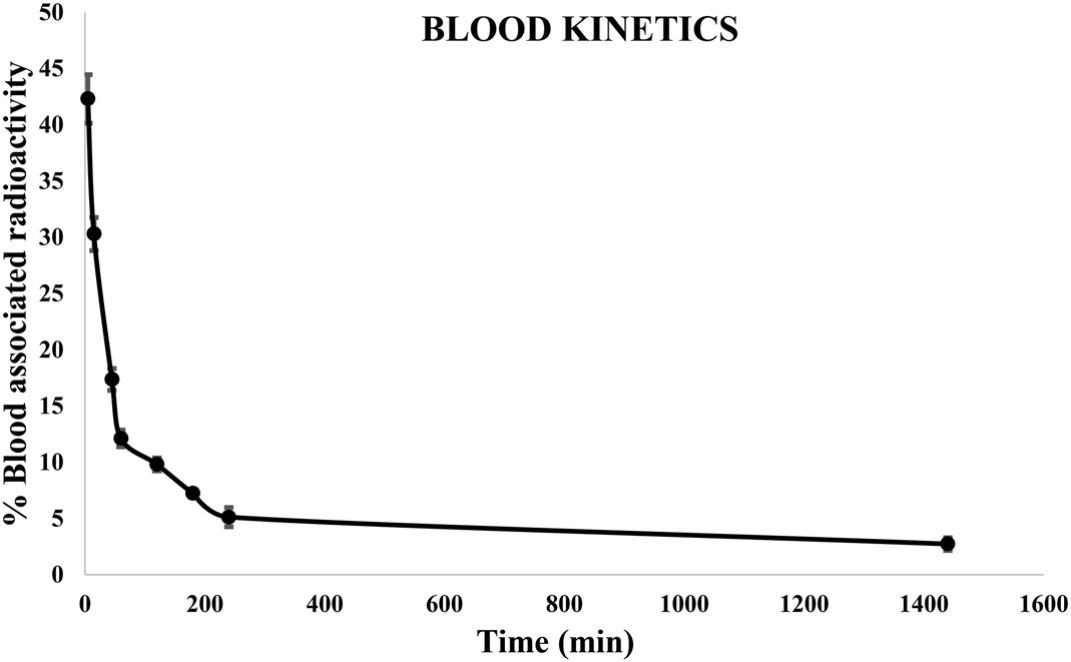
Blood kinetics of ^99m^Tc-labelled MC in rabbit: 42% of Tc-99m labelled muscarine reached the blood pool.

**Figure 20:**
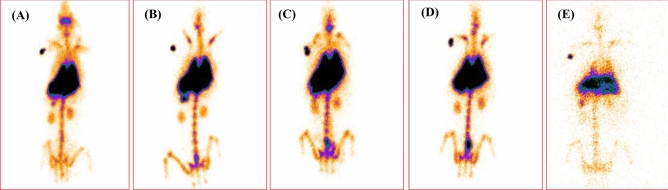
Gamma Scintigraphy of Rabbit which was administered ^99m^Tc-labelled MC via intravenous route (**A**) 5 min, (**B**) 1 h, (**C**) 2 h, (**D**) 4 h, (**E**) 24 h.

The blood pharmacokinetics showed that approximately 42% of Tc-99m labelled muscarine reached the blood pool within 5 min after administration (Fig. 19). As indicated previously by the early onset of muscarinic symptoms in mice, this finding reiterates that muscarine is a fast acting toxin. The biodistribution pattern noted immediately after administering muscarine (Fig. 20A) indicated that the major proportion of muscarine that diffused into the organs was concentrated in the thorax region (lungs, heart, liver and stomach) of the animal (Supplementary Table. S1). It was observed that muscarine was also distributed to the regions of the upper body and this observation correlated well with the appearance of characteristic muscarinic symptoms such as miosis (constriction of the pupils) and hypersalivation (activation of the salivary glands) in the animals within 10–15 min post administration (relative percentage of biodistribution as shown in the Supplementary Table. S1). The concentration of muscarine in blood gradually declined to 30% and 17% after 15 and 45 min respectively and reached to about 12.11% by the end of 1 h (Fig. 19). The biodistribution at this point (Fig. 20B) indicated the presence of muscarine in spleen, besides the organs noted earlier. The blood concentration further declined to 9.8% at the end of 2 h and the biodistribution study recorded the presence of muscarine in bladder (Fig. 20C) suggesting the beginning of renal clearance of muscarine. The fall in concentration of muscarine in blood reached 5.11% by 4 h (Fig. 20D) and at the end of 24 h, only 2.72% of muscarine was noted in circulation. Gamma scintigraphy clearly indicated that the residual muscarine in the body was deposited in the liver (Fig. 20 E).

Thus, direct radiolabeling using technetium-99m was found useful to study the biodistribution of muscarine. Significant amount of the toxin was quickly and effectively concentrated in the thorax and head region. This study closely explains the early muscarinic response such as miosis and salivation in mice. Also, a relatively major proportion of the muscarine administered was accumulated in the liver by the end of 24 h which possibly could have contributed to the hepatotoxicity of *Inocybe virosa* (Results unpublished).

## Conclusion

The potential risks involved in consuming *Inocybe virosa* has been clearly indicated by its in vitro and in vivo toxicological evaluation. The analysis of the volatile profile of the extract indicated the presence of potentially toxic compounds such as methyl palmitate, phenol, 3,5-bis(1,1-dimethyl ethyl). However, their role in toxicity of *Inocybe virosa* has to be verified. This study also identified muscarine as one of the major toxin in *Inocybe virosa* as indicated by HPLC analysis and further confirmed by LC–MS analysis. This was further substantiated by the bioassay which showed that the extract had comparable effects of an equivalent dose of muscarine. The biodistribution study of muscarine pointed out that liver was one of the target organs of muscarine where it persisted upto 24 h.

## Supplementary information

Supplementary information.
