# Supplementary material for: Toxic metabolite profiling of Inocybe virosa
Source: Sci Rep. 2020 Aug 13;10:13669. doi: 10.1038/s41598-020-70196-7 (PMC7426861; doi:10.1038/s41598-020-70196-7)
Supplement: Supplementary file 1 — Supplementary information. [file 41598_2020_70196_MOESM1_ESM.docx]

# Toxic metabolite profiling of *Inocybe virosa*

# Sai Latha S*, Naveen S*, Mahadeva Naika*, Anilakumar K R*

# Ankur Kaul^#^, Gaurav Mittal^#^

* Defence Food Research Laboratory, Defence Research and Development Organisation, Mysore-570011, Karnataka, India

^#^ Institute of Nuclear Medicine and Allied Sciences, Defence Research and Development Organisation, New Delhi-110054, India

**Running Title: Toxic metabolites of *Inocybe virosa***

CORRESPONDING AUTHOR

**Dr Naveen S**

Scientist D

Department of Food Quality and Assurance

[Defence Food Research Laboratory](http://www.drdo.gov.in/drdo/labs/DFRL/English/index.jsp?pg=homebody.jsp)

[Defence Research and Development Organisation](http://www.drdo.gov.in/drdo/English/index.jsp?pg=homebody.jsp)

Ministry of Defence, Govt. of India,

Siddarthanagar, Mysore-570011, INDIA

Ph:9964179904

e-mail: [naveens.dfrl@gmail.com](mailto:naveens.dfrl@gmail.com)

Supplementary figures

Supplementary Figure S1

HPLC Chromatogram of Muscarine standard

1. 0.1 µg
2. 0.2 µg
3. 0.3 µg
4. 0.4 µg


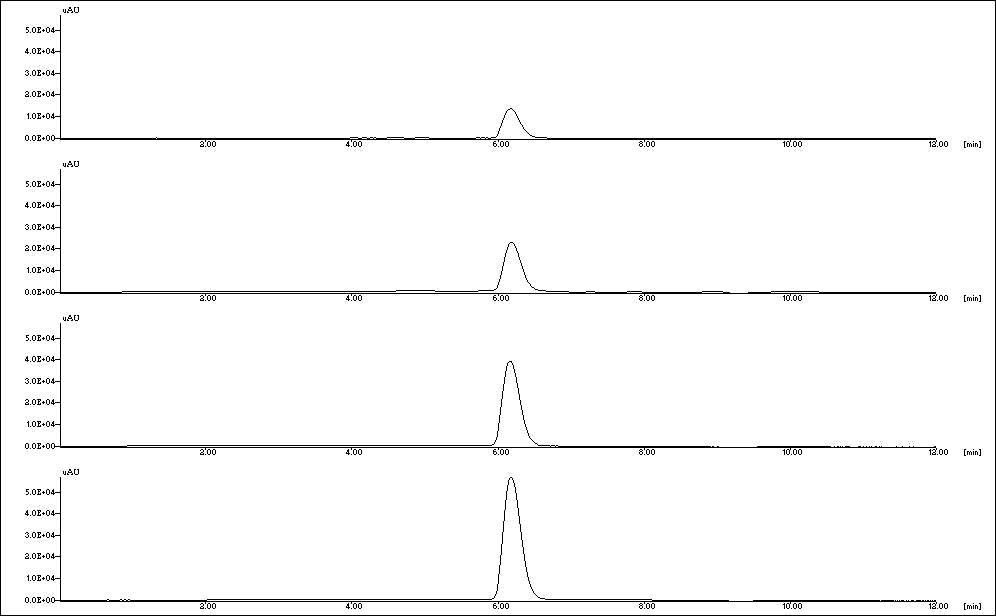


A

B

C

D

The above figure is regarding the RP-HPLC of Muscarine standard procured from Sigma. This chromatogram was used for the analysis of *Inocybe virosa* for the toxin muscarine’s presence.

**Supplementary figures**

**Supplementary Figure S1**

**Biodistribution of ^99m^Tc labelled MC in rabbit (in % of whole body deposition)**

| **ORGAN/TISSUE** | **5 MIN** | **1 HR** | **2 HR** | **4 HR** | **24 HR** |
| --- | --- | --- | --- | --- | --- |
| **HEAD REGION** | 6.09 ± 0.17 | 1.23 ± 0.40 | 1.02 ± 0.47 | 0.33 ± 0.13 | 0.94 ± 1.54 |
| **NECK REGION** | - | - | - | 0.54 ± 0.12 | 0.04 ± 0.04 |
| **THORAX REGION** | 71.72 ± 0.62 | 62.21 ± 1.54 | 45.53 ± 1.67 | 37.40 ± 2.44 | - |
| **LUNG** | - | - | - | - | 0.40 ± 0. 53 |
| **LIVER** | - | - | - | - | 2.82 ± 1.89 |
| **STOMACH** | - | - | - | - | 0.74 ± 1. 65 |
| **SPLEEN** | - | 1.39 ± 0.02 | 3.07 ± 0.09 | - | 0.10 ± 0.17 |
| **L.KIDNEY** | 0.91 ± 0.13 | 0.44 ± 0.05 | 0.38 ± 0.03 | 0.34 ± 0.02 | 0.03 ± 0.03 |
| **R.KIDNEY** | 1.15 ± 0.78 | 0.49 ± 0.50 | 0.42 ± 0.25 | 0.47± 0. 26 | 0.07 ± 0.42 |
| **BLADDER** | - | - | 1.03 ± 0. 80 | 1.84 ± 0.34 | 0.10 ± 0.44 |

The above table explains the distribution of radio labelled MC in rabbit in the whole body.
